# Study on amniotic fluid metabolism in the second trimester of Trisomy 21

**DOI:** 10.1002/jcla.23089

**Published:** 2019-11-10

**Authors:** Xiaoting Liu, Sheng Quan, Yurong Fu, Weiwei Wang, Wenling Zhang, Xiaofei Wang, Chenxi Zhang, Daijun Xiang, Liwen Zhang, Chengbin Wang

**Affiliations:** ^1^ Medical School of Chinese PLA & Medical laboratory center First Medical Center of Chinese PLA General Hospital Beijing China; ^2^ Hangzhou Calibra Diagnostics, LTD. Hangzhou China

**Keywords:** amniotic fluid, biomarkers, metabolism, metabolomics, trisomy 21

## Abstract

**Background:**

Trisomy 21 is a common aneuploid condition in humans and accounts for approximately one quarter of all aneuploid live births. To date, early diagnosis of Trisomy 21 remains a challenging task. Metabolomics may prove an innovative tool to study the early pathophysiology of Trisomy 21 at a functional level.

**Methods:**

Ultra‐performance liquid chromatography coupled with mass spectrometer (UPLC‐MS) was used for untargeted metabolomic analysis of amniotic fluid samples from women having normal and trisomy 21 fetuses.

**Results:**

Many significantly changed metabolites were identified between amniotic fluid samples from Trisomy 21 pregnancies and normal euploid pregnancies, such as generally lower levels of several steroid hormones and their derivatives, higher levels of glutathione catabolites coupled with lower levels of gamma‐glutamyl amino acids, and increased levels of phospholipid catabolites, sugars, and dicarboxylic acids. The identification of a human milk oligosaccharide in amniotic fluid may worth further investigation, since confirmation of this observation may have significant implications for regulation of fetal development.

**Conclusions:**

The metabolisms in amniotic fluid from Trisomy 21 and normal pregnancies are quite different, and some of the significantly changed metabolites may be considered as candidates of early diagnostic biomarkers for Trisomy 21.

## 1 INTRODUCTION

Trisomy 21 (T21), also known as Down Syndrome, is a common aneuploid condition in humans and accounts for approximately one quarter (approximately 0.13%) of all aneuploid live births.^1^, ^2^ The medical condition of T21 can lead to varying degrees of physical and mental malformations that are characterized by low intelligence, stunted physical development, and distinctive facial features.^3^, ^4^ Serological triple screening (including AFP, beta–HCG, and estriol) combined with amniocentesis has become one of the main methods for prenatal diagnosis of chromosome diseases such as Trisomy 21.^5^, ^6^ Early diagnosis of T21 pregnancies can give both the expecting parents and doctors the benefit of early awareness and preparation for dealing with the difficult situation.

Amniotic fluid (AF) is the biological fluid in the amniotic sac surrounding a fetus in the mother's womb. Its volume and composition is dynamically affected by fetal lung secretion, and urine, as well as nutrients/water exchanges between the mother and fetus through membranes.^7^ Amniotic fluid has very low levels of proteins and enzymes in early pregnancy (<11 weeks), but in mid‐ to late pregnancy the protein levels in amniotic fluid increase, and the fluid accumulates many metabolites from the fetal metabolism due to free circulation from the fetal respiratory and digestive tracks.^8^ Since amniotic fluid reflects metabolisms of both the mother and the fetus, understanding of its composition is important for early investigation of a wide spectrum of clinical conditions.

Metabolomics is one of the ‐omics approaches that studies small metabolic molecules in biological samples. It can provide a comprehensive picture of metabolic responses of an organism to pathophysiological stimuli. Metabolomics study result may serve as an excellent reflection of an organism's phenotypes and reveal molecular signatures of certain disease states or physiological conditions. Commonly used techniques for metabolomics studies include nuclear magnetic resonance (NMR) spectroscopy and mass spectrometry (MS), which can detect various kinds of metabolites such as amino acids, oligopeptides, sugars, steroids, biliary acids, fatty acids, and other intermediary metabolic compounds.

Metabolomics studies of amniotic fluid have been reported for GDM of pregnant women,^9^ the prediction of preterm labor,^10^, ^11^ and deformity.^12^, ^13^ Amniotic fluid collected in the second trimester of pregnancy was studied for prenatal disorders using NMR spectroscopy to search for metabolite biomarkers of preterm delivery.^9^ Ultra‐performance liquid chromatography coupled with mass spectrometry (UPLC‐MS) was used to investigate the effects of preterm delivery on amniotic fluid metabolisms during the second trimester. In the preterm delivery group, the decreases of certain amino acids and increase of a hexose (possibly glucose) suggest alterations in placental amino acid fluxes and possibility of hyperglycemia.^13^ Proton NMR spectroscopy was performed on human amniotic fluid samples representing pregnancies at different maturation stages and with different fetal‐maternal complications.^14^ In another study of T21 amniocytes using matrix‐assisted laser desorption/ionization time‐of‐flight mass spectrometry (MALDI‐TOF), six proteins (calumenin, nucleophosmin, elongation factor 1‐beta, cathepsin D, platelet‐activating factor acetylhydrolase IB subunit beta, and 14‐3‐3 protein beta/alpha) were found to be significantly accumulated.^15^ Alterations in metabolic pathways of porphyrin metabolism, bile acid metabolism, hormone metabolism, and amino acid metabolism were discovered in amniotic fluid from fetuses with Down syndrome using UPLC‐TOF‐MS.^16^

The abovementioned studies have only identified limited numbers of metabolites in amniotic fluid, and how to discover as many amniotic fluid metabolites as possible remains a challenge, especially with T21 amniotic fluid samples which have been less studied. The aim of this study was to explore potential biomarkers of T21 in amniotic fluid of second trimester using an untargeted metabolomics approach and try to understand the underlying metabolic mechanisms of T21 birth defect. Ultra‐performance liquid chromatography coupled with mass spectrometry (UPLC‐MS) was utilized to identify significantly changed metabolites between T21 and normal amniotic fluid samples.

## 2 MATERIALS AND METHODS

### 2.1 Study population

This study was approved by the First Medical Center of Chinese PLA General Hospital. Amniotic fluid samples were collected from 699 pregnant women with singleton pregnancies between March 2018 and March 2019. Each participant of the study has provided written informed consent. The amniotic fluid samples were collected from the participants when they were undergoing amniocentesis for routine clinical indications including advanced maternal age, abnormal quadruple/triple test, family history of chromosomal abnormalities, suspected fetal anomalies or infection, and maternal request during the second trimester of gestation. The clinical test results from ultrasonography, karyotyping, and CMA were also collected. Pregnancies with obstetric complications including hypertension diabetes, premature rupture of the membranes, and uterine infections were excluded from the study. The case group of this study includes 21 amniotic fluid samples of normal pregnancies with DS fetuses, and the control group contains 21 amniotic fluid samples of normal pregnancies with healthy fetuses. Samples in the case and control groups are matched upon maternal age, gestational age, and fetal gender in a 1:1 ratio.

### 2.2 Amniotic fluid samples

Transabdominal amniocentesis was performed with a 21‐gauge needle under ultrasound guidance to evaluate the position of the fetus. The total of 5 mL residual AF was collected. The collected AF Samples were transported immediately in a capped sterile syringe to the biobank of the First medical center of Chinese PLA General Hospital. At the biobank, the AF samples were centrifuged for 10 minutes at 500 *g* at 4°C, and the supernatants were recovered, aliquoted, and stored at −80°C. The total processing time was <4 hours from the time of sample collection to the time of sample freezing.

### 2.3 Sample preparation for metabolomics study

The amniotic fluid samples were processed using an automated MicroLab STAR^®^ liquid handling system from Hamilton Company. Several recovery standards were added prior to the first step in the extraction process to evaluate the efficiency of sample extraction. Each sample was extracted with methanol under vigorous shaking for 2 minutes in a SpexSamplePrep (USA) GenoGrinder 2010 tissue homogenizer to precipitate proteins and dissociate small molecule metabolites bound to proteins or trapped in the precipitated protein matrix. The vortexed extraction mixture was then centrifuged, and the supernatant was collected to recover chemically diverse metabolites. The resulting extract was divided into five fractions: two for analysis by two separate reverse‐phase (RP)/UPLC‐MS/MS methods using positive ion mode electrospray ionization (ESI), one for analysis by RP/UPLC‐MS/MS using negative ion mode ESI, one for analysis by HILIC/UPLC‐MS/MS using negative ion mode ESI, and one was reserved for backup. Each fraction was dried under nitrogen gas to remove the organic solvent, and the dried fraction was reconstituted in solvents compatible with each of the four UPLC‐MS methods.

### 2.4 uplc‐ms analysis

The UPLC‐MS analysis utilized a Waters ACQUITY ultra‐performance liquid chromatography (UPLC) and Thermo Scientific Q‐Exactive high resolution/accurate mass spectrometer which is interfaced with a heated electrospray ionization (HESI‐II) source and an orbitrap mass analyzer operated at 35 000 mass resolution. Each sample contains a series of added chemical standards at fixed concentrations to ensure sample injection and chromatographic consistency. For the four aliquots of each extracted sample, the first aliquot is analyzed by liquid chromatography optimized for more hydrophilic compounds under positive ionization. In this method, the sample was gradient‐eluted from a C18 reverse‐phase column (Waters UPLC BEH C18‐2.1 × 100 mm, 1.7 µm) using water/methanol mobile solutions containing 0.05% perfluoropentanoic acid (PFPA) and 0.1% formic acid (FA). The second aliquot was also analyzed under positive ionization, but the liquid chromatography is optimized for more hydrophobic compounds. In this method, the extract is gradient‐eluted from the aforementioned C18 reverse‐phase column using methanol/acetonitrile/water mobile solutions containing 0.05% PFPA and 0.01% FA. The third aliquot is analyzed under negative ionization mode. Each sample is gradient‐eluted from a C18 column using methanol/water mobile solutions containing 6.5 mmol/L ammonium bicarbonate at pH 8. The fourth aliquot is analyzed under negative ionization mode after the injection is gradient‐eluted from a HILIC column (Waters UPLC BEH Amide 2.1 × 150 mm, 1.7 µm) using mobile solutions containing water/acetonitrile with 10 mmol/L ammonium formate at pH 10.8. The MS analysis in the QE orbitrap alternates between MS and data‐dependent MS^n^ scans using dynamic exclusion.

### 2.5 Data pretreatment and chemometric analysis

After evaluation and passing the quality control (QC) inspection, the raw data are extracted and peak‐identified using the proprietary IT hardware and software developed by Metabolon, which are built on a web‐service platform utilizing Microsoft's NET technologies, high‐performance application servers, and fiber channel storage arrays in clusters to provide active failover and load balancing. Compounds are identified by comparing experimental features to library entries of purified compound standards or recurrent unknown entities. The library is based on authenticated standards and contains the retention time/index (RI), mass to charge ratio (*m/z)*, and MS/MS spectral data of all standards present in the library. A definite identification is based on three criteria: retention index within a narrow RI window of the proposed identification, accurate mass match to the library with ± 10 ppm, and good MS/MS forward and reverse scores. MS/MS scores are based on a comparison of the ions present in the experimental spectrum to ions present in the library entry spectrum. The use of all three criteria enables distinction and accurate identification of similar compounds. More than 4500 commercially available pure standard compounds have been acquired and registered into LIMS for analysis on all four platforms for determination of their analytical characteristics. Additional mass spectral entries have been created for structurally unnamed compounds, which have been identified by virtue of their recurrent nature (both chromatographic and mass spectral).

For univariate statistical analysis, missing values were filled with the minimum observed value for each compound, and the data were transformed to the natural log for Welch's *t* test to identify metabolites that differed significantly between experimental groups. Multi‐dimensional analyses including PCA, HCA, and Random Forest were performed using the R package.

## 3 RESULTS

### 3.1 Patients characteristics

A total of 21 cases of fetal T21 and 21 controls were recruited in our study. The average maternal age of both the cases and controls is 34 years old; the average gestational age is 132 days for cases and 133 days for controls, respectively. The fetal genders were 11 males and 10 females in both groups (Table1).

**Table 1:** Demographic characteristics of patients

Characteristics	T21	Control
Number of samples	21	21
Maternal ages (y)	34 (24‐42)	34 (26‐41)
Gestational ages (d)	132 (110‐142)	133 (112‐141)
Fetal gender	11 male, 10 female	11 male, 10 female

### 3.2 Metabolomics study results

A total of 621 compounds of known identity were discovered from the amniotic fluid samples through the UPLC‐MS/MS metabolomics platform. Welch's two‐way *t* tests were performed to identify metabolites that differed significantly between the case and control groups, and 151 metabolites were found to be significantly different between the two groups. Among the 151 metabolites, 73 are upregulated and 78 downregulated in the T21 case group (Table 2).

**Table 2:** Number of significantly changed metabolites in T21 AF

Ratio	Number of significantly changed metabolites (*P* < .05)	Up/Down
Disease/Control	151	73/78

### 3.3 Principle component analysis

**Figure 1:**
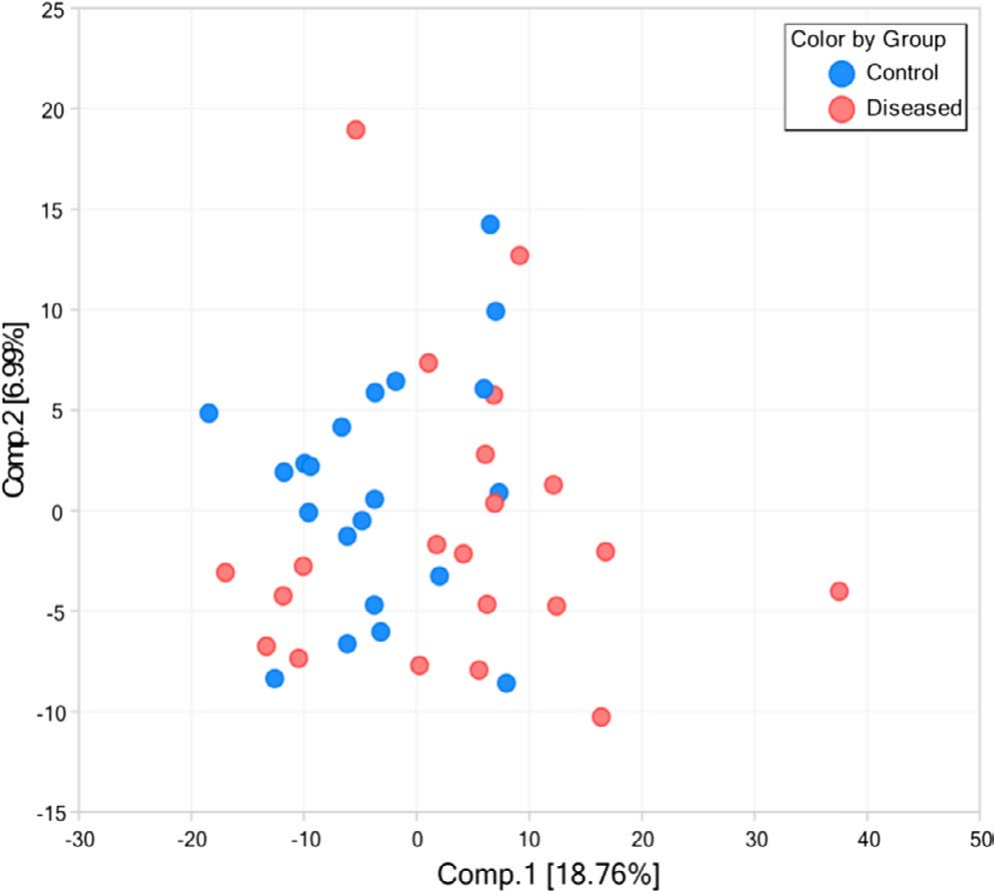
Principal component analysis

As shown in Figure 1, while the Principal Component Analysis (PCA) does not yield a clear separation of the case and control groups, there is a slight tendency for control samples to cluster along component 1 (which accounts for 19% of the variance), and for the T21 samples to occupy the more extreme values in the plot, suggesting that T21 samples differ from the controls in multiple fashions, rather than changing in a uniform and predictable manner.

### 3.4 Hierarchical cluster analysis

**Figure 2:**
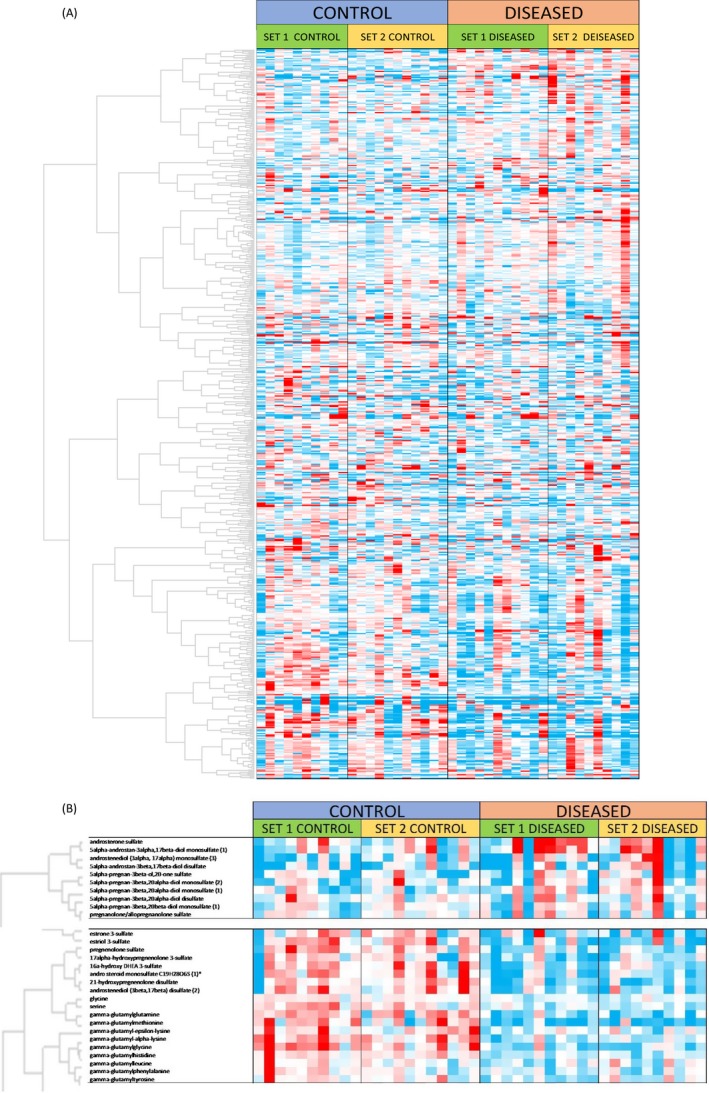
A, Hierarchical cluster analysis. B, Hierarchical clusters of steroid hormones and gamma‐glutamyl amino acids

Broad individual sample variation in the data is displayed in the hierarchical cluster (HC) heat map plot (Figure 2A). Despite this large interindividual biological variance, which is very common in human biological samples, the HC plot is a valuable method for identification of compounds having significant correlations within the dataset. For example, many compounds common to particular pathways, including steroid hormones and a group of *gamma‐*glutamyl derivatives of amino acids, are clustered together (Figure 2B).

### 3.5 Random forest analysis

**Figure 3:**
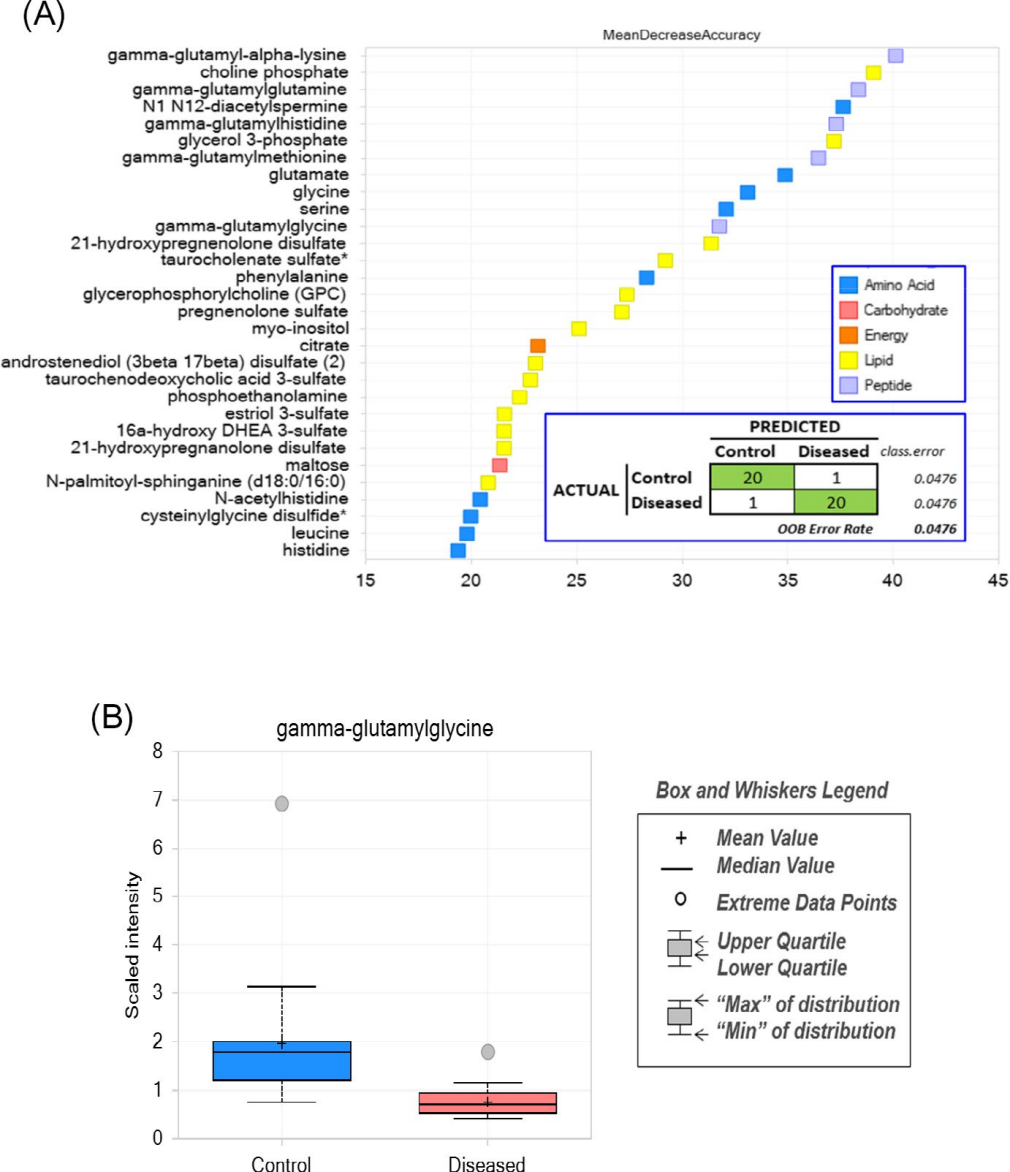
A, Random forest analysis. B, Gamma‐glutamylglycine scaled intensity in amniotic fluid between control and disease group

Random Forest (RF) analysis is a supervised learning algorithm testing how accurately the obtained metabolite data can assign each sample into the proper experimental group. As shown in Figure 3, Random Forest Analysis of the amniotic fluid metabolomics data is able to classify 95% of the control and case samples correctly. Most of the metabolites responsible for this fairly accurate classification fall into four general metabolic pathway categories: the *gamma*‐glutamyl amino acids, the steroid hormone derivatives, polyamines (N1, N12‐diactylspermine), and glycerol derivatives resulting from degradation of phospholipids. The steroid hormones and the *gamma*‐glutamyl amino acids are mostly lower in the T21 case amniotic fluids relative to the controls, while N1, N12‐diacetylspermine, and the phospholipid derivatives are higher in the T21 group.

### 3.6 Significantly changed metabolites in T21 amniotic fluid

**Figure 4:**
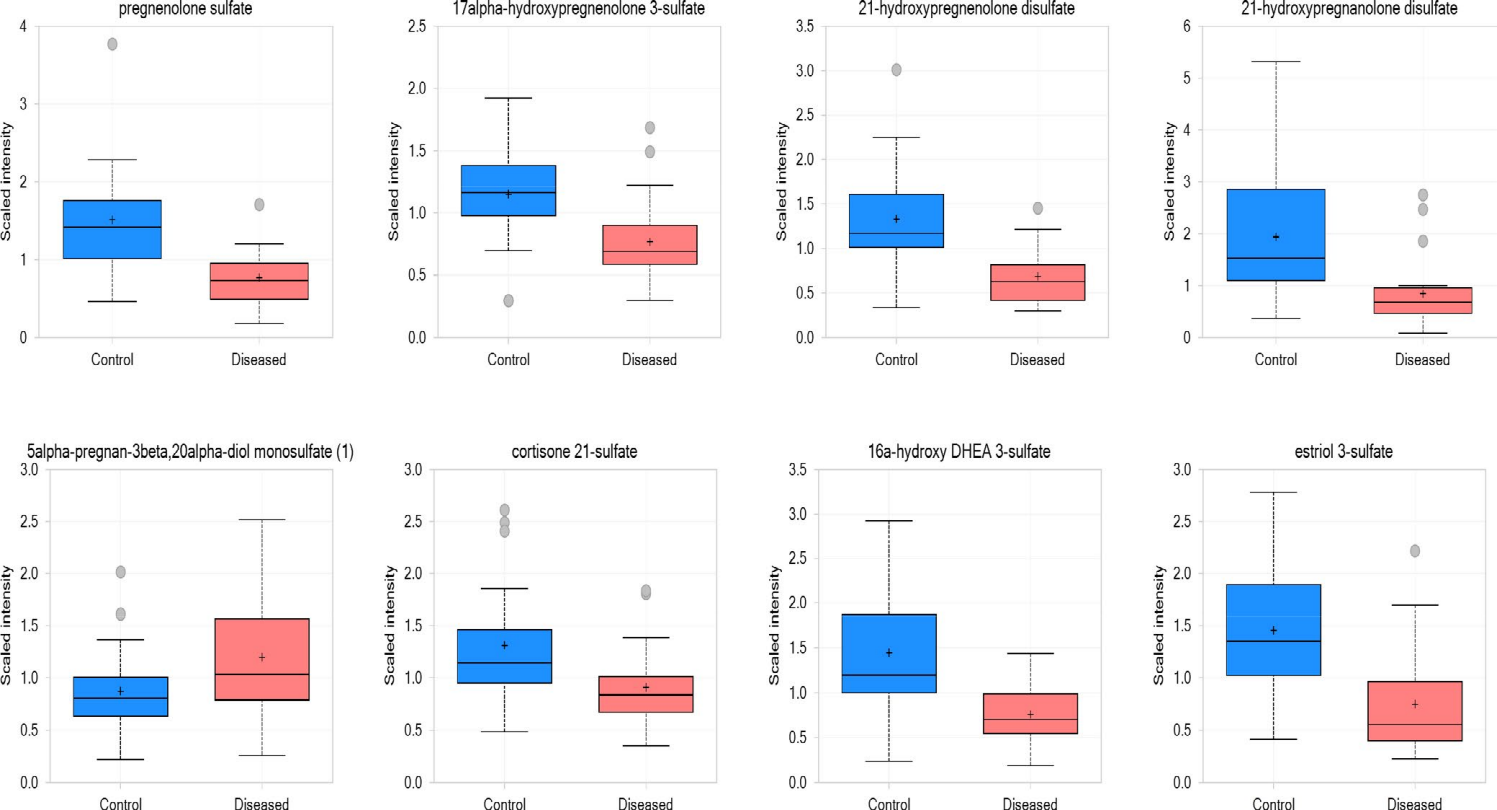
Box plots showing different levels of steroid hormones between the control and disease group

**Figure 5:**
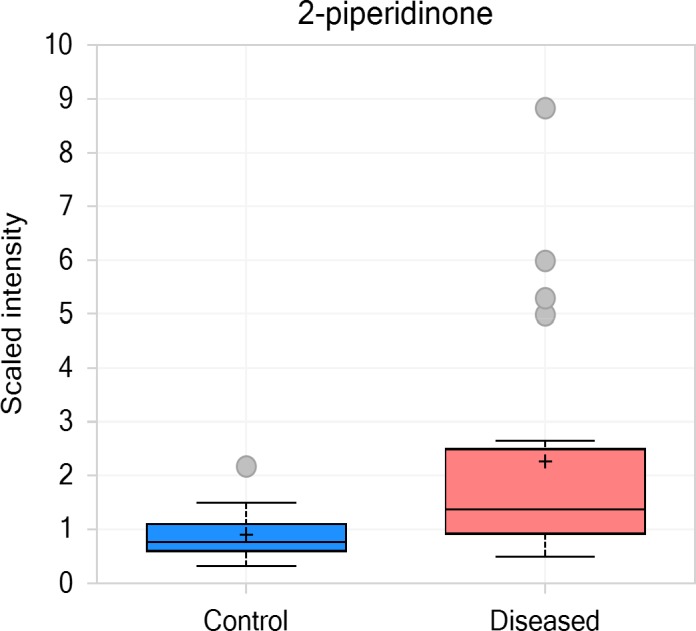
*Delta*‐lactam compound 2‐piperidinone in the control and disease group

Comparing to their levels in the control group, 13 of the 20 identified steroids hormones are significantly changed in T21 amniotic fluid with 11 decreasing and 2 increasing (Table 3, Figures 4 and 5). The decreased steroids hormones in T21 amniotic fluid include pregnenolones, corticosteroids, androgens, and estrogens. The progestin class compound 5*alpha*‐pregnan‐3*beta* 20*alpha*‐diol monosulfate is significantly increased in T21 amniotic fluid (*P* = .054).

**Table 3:** Significantly changed metabolites between the control and disease group

Sub pathway	Biochemical name	Disease/Control
Gamma‐glutamyl amino acid	gamma‐glutamylglutamine	0.47
	gamma‐glutamylglycine	0.39
	gamma‐glutamylhistidine	0.62
	gamma‐glutamylisoleucine	1.02
	gamma‐glutamylleucine	0.81
	gamma‐glutamyl‐alpha‐lysine	0.48
	gamma‐glutamyl‐epsilon‐lysine	0.65
	gamma‐glutamylmethionine	0.31
	gamma‐glutamylphenylalanine	0.72
	gamma‐glutamylthreonine	0.89
	gamma‐glutamyltyrosine	0.73
	gamma‐glutamylvaline	0.85
Phospholipid metabolism	choline phosphate	1.84
	glycerophosphorylcholine (GPC)	1.79
	phosphoethanolamine	1.85
	glycerophosphoethanolamine	1.24
	glycerophosphoserine	1.13
	glycerophosphoinositol	2.11
	trimethylamine N‐oxide	1.33
Fatty acid, dicarboxylate	glutarate (C5‐DC)	1.26
	2‐hydroxyglutarate	1.16
	adipate (C6‐DC)	1.05
	2‐hydroxyadipate	1.36
	3‐hydroxyadipate	1.35
	suberate (C8‐DC)	1.42
	sebacate (C10‐DC)	1.01
	decadienedioic acid (C10:2‐DC)	1.48
	azelate(C9‐DC)	1.19
Pentose metabolism	ribitol	1.2
	ribonate	1.27
	arabitol/xylitol	1.15
	arabonate/xylonate	1.28
	sedoheptulose	1.26
	ribulonate/xylulonate/lyxonate	1.36
Glycogen metabolism	maltose	1.81
Disaccharides and oligosaccharides	lactose	0.86
	3′‐sialyllactose	1.24
	sucrose	2.25
Fructose, mannose, and galactose metabolism	fructose	1.04
	mannitol/sorbitol	1.3
	mannose	0.99
	galactonate	1
Aminosugar metabolism	glucuronate	0.98
	N‐acetylneuraminate	1.31
	erythronate	1.11
	N‐acetylglucosamine/N‐acetylgalactosamine	0.9
TCA cycle	citrate	1.28
	aconitate [cis or trans]	1.23
	isocitrate	1.22
	alpha‐ketoglutarate	0.84
	succinylcarnitine (C4‐DC)	0.99
	succinate	0.86
	fumarate	0.86
	malate	0.85
	2‐methylcitrate/homocitrate	1.12

## 4 DISCUSSION

Not many metabolomics studies have been conducted on fetus abnormalities such as trisomy 21, especially on amniotic fluid samples. In a few metabolomics studies that have been conducted on amniotic fluids, only a limited number of metabolites have been identified.^9^, ^12^, ^13^, ^14^ Using a highly quality‐controlled metabolomics platform consisting of four specific UPLC‐MS/MS methods targeting different groups of metabolites, we identified 621 metabolites in amniotic fluid samples, and 151 of the 621 metabolites are discovered to be significantly different between the T21 and normal pregnancy amniotic fluids. The clustering together of many metabolites that belong to the same metabolic pathways in the hierarchical cluster analysis (such as steroid hormones and a group of *ga*mma‐glutamyl derivatives of amino acids) indicates the reliability of the metabolomics platform we used.

Elevated prenatal cortisol has been associated with several negative conditions in infancy, including aborted fetuses, excessive fetal activity, delayed fetal growth/development, prematurity, low birth weight, and attention/temperament problems.^17^ Compared with the control, 13 of the 20 identified steroids hormones are significantly changed in T21 amniotic fluid with 11 decreasing and 2 increasing. Similarly, two hormones have been reported to be significantly altered in T21 amniotic fluid.^16^ In humans, analysis of total urinary steroids was effective in detecting fetuses with Smith‐Lemli‐Opotz syndrome,^18^ and progesterone levels were altered in 87% of maternal urine in the presence of a DS fetus.^19^ Increased levels of cortisol and decreased levels of pregnenolone sulfate were also detected in DS fetuses, which may be associated with abnormal bone and brain development.^19^ Steroid hormones are important in a variety of physiological processes, including development, sexual dimorphism, neural function, osmoticbalance, and stress responses.^20^ Many steroid hormones are involved in fetal development, and their levels change greatly throughout fetus development until birth. Their levels in amniotic fluid are affected by interactions of liver and adrenal glands functions of both the mother and fetus through the placenta.^21^ The medical significance of generally lower steroid hormones in Trisomy 21 amniotic fluid is not clear, but other aneuploid and mutation events which can affect hormone synthesis and lead to developmental symptoms have been reported.^22^, ^23^ Because hormone levels fluctuate strongly during development as well as affected by maternal age,^24^ we cannot rule out the possibility that these observed changes of steroid hormones in T21 reflect the kinetics of fetal maturation process.

Free amino acids levels were mostly lower in T21 amniotic fluid relative to the controls. In a previous study reported by Huang et al,^16^ the authors found that the T21 amniotic fluid contains less arginine, histidine, and glutamate than the regular amniotic fluids. These findings are also confirmed in our study. Other than these 3 amino acids, more are found to be decreased in our study, including glycine, serine, aspartate, asparagine, phenylalanine, tyrosine, tryptophan, leucine, isoleucine, and methionine. A group of *gamma*‐glutamyl amino acids including gamma‐glutamylglutamine, gamma‐glutamylglycine, gamma‐glutamylhistidine, gamma‐glutamylleucine, gamma‐glutamyl‐alpha‐lysine, gamma‐glutamyl‐epsilon‐lysine, gamma‐glutamylmethionine, gamma‐glutamylphenylalanine, and gamma‐glutamyltyrosine are all lower in T21 amniotic fluid samples. Gamma‐glutamyl amino acids are involved in glutathione metabolism, while it is possible that the lower *gamma*‐glutamyl amino acid levels simply reflect the lower amino acid pools, it is also noteworthy that one of the strongest biomarkers for predicting the T21 state in RF analysis is cysteinylglycine disulfide (3.4‐fold higher), which is a glutathione catabolite. Glutathione and its catabolites are important for the remediation of oxidative stress, and thus, a disruption of their biosynthetic and salvage cycle could reflect a stressful physiological condition in T21 amniotic fluid. The *gamma‐*glutamyl derivatives of amino acids are formed in humans by the enzyme *gamma*‐glutamyltranspeptidase (GGT), an enzyme well known to be affected in its expression during aneuploid pregnancies.^8^ Significant reduction of GGT activity in amniotic fluid from pregnancies with Trisomy 21 has been reported.^25^

The T21 amniotic fluid samples contain significantly higher levels of phospholipid catabolites, namely the glycerol backbones and head groups resulting from lipolysis by phospholipases A, C, and D. Amniotic fluid from several abnormal pregnancy types (prematurity, respiratory distress syndrome, hyaline membrane disease, anencephaly with polyhydramnios) has been shown to contain lowered levels of intact phospholipids.^26^ Elevated phospholipase activity might be expected to reduce all the phospholipid species (assuming constant phospholipids production rates), but all phospholipid species detected in this study (mostly phosphatidylcholines) do not show statistically significant reductions in T21 amniotic fluid. It is possible that the higher phospholipid catabolite levels just reflect increased lipolysis within specific fetal organs, such as in the kidney where phospholipids, especially glycerophosphocholine (GPC), can serve important osmolyte functions.^27^

Several short‐ and mid‐chain dicarboxylic acids are found to be significantly higher in the case samples. These compounds are formed by *omega* oxidation of short‐ and mid‐chain fatty acids. They have been found to be elevated in amniotic fluid of pregnancies in which fetuses are affected by glutaric aciduria type II, a genetic disorder that can lead to brain malformations, enlarged liver, and other developmental problems.^28^ Glutaric aciduria type II can result from deficiencies in one of three proteins, the *alpha* (ETFA) and *beta* (ETFB) subunits of electron transfer flavoprotein, and electron transfer flavoprotein dehydrogenase (ETFDH).^29^ It is not known if Trisomy 21 may affect the expression of one or more of these functionalities.

The T21 amniotic fluid samples contain significantly higher levels of N1,N12‐diacetylspermine comparing to its level in the normal pregnancy amniotic fluid. N1,N12‐Diacetylspermine is a polyamine commonly found in human urine.^30^ It has been reported that urinary N1,N12‐Diacetylspermine can be used as a marker to efficiently detect colorectal and breast cancers at early stages.^31^ Whether N1, N12‐Diacetylspermine has any such function at the fetal stage is not yet known. Its presence in amniotic fluid raises the possibility that its influences on fetal development may be occurring even before birth. We could not find any evidence in the literature that anyone has investigated the presence of N1‐N12‐Diacetylspermine in amniotic fluid.

Several pentose derivatives, as well as maltose and 3′‐sialyllactose (3′‐SL), are also found to be increased in T21 amniotic fluid in this study. To our knowledge, the reason for these differences in sugars is not clear, but dietary profile differences between the case and control groups (if such differences exist) might account for the observations. The fact that citrate, aconitate, and isocitrate are all higher while all the other compounds in the TCA cycle are lower in T21 samples is consistent with a known mitochondrial defect in trisomy 21, in which several mitochondrial enzymes, including isocitrate dehydrogenase, show reduced activities.^32^ It was interesting, but unexpected, that lactose and 3′SL are detected in all amniotic fluid samples (case and control). To confirm these observations would require measuring them with an orthogonal technique, using sensitive targeted assays, and ideally with absolute quantitation (eg, using isotopic standards). If confirmed by targeted assay, it would indicate that milk sugars in maternal mammary glands can make their ways to the fetus. Human milk oligosaccharides (HMOs) like 3′SL are known to promote gut maturation and immunomodulary functions in newborns,^33^ but whether they have any function at the fetal development is not known. At present, we could not find any study that has reported the presence of HMOs in amniotic fluid.

Other interesting discoveries include a large increase (2.5‐fold) of 2‐piperidinone, a delta‐lactam compound, in the T21 amniotic fluid. This compound is typically a xenobiotic, possibly of maternal dietary origin, but it can also be made through enzymatic oxidation and cyclization of cadaverine in mouse liver,^34^ suggesting possible perturbations of fetal liver functions.

## 5 CONCLUSIONS

In summary, the current metabolomics study identifies the largest number of metabolites in amniotic fluids (a total of 621 named metabolites), and many of them are significantly changed in the Trisomy 21 amniotic fluids. These differences include generally lower levels of several steroid hormones and their derivatives, higher levels of glutathione catabolites coupled with lower levels of many gamma‐glutamyl amino acids, and increased levels of phospholipid catabolites, sugars, and dicarboxylic acids. The identification of a human milk oligosaccharide in amniotic fluid may worth further investigation since confirmation of this observation may have significant implications for regulation of fetal development. The large number of discovered metabolite changes greatly expands the scope of our understanding of T21amniotic fluid metabolism. While confirming and discovering (but discovering more) more changes in amino acids and hormones that have been reported previously,^16^ we have also discovered new changes in polyamine metabolites, sugar metabolites, and metabolites related to liver damage.
